# The complete chloroplast genome of *Tetrastigma planicaule*, one important folk medicinal plant in China

**DOI:** 10.1080/23802359.2021.1930598

**Published:** 2021-05-24

**Authors:** Xin Huang, Qihua Zhou, Cimei Qin, Chun Mao, Kaidao Sun, Bo Qin, Qing Tang

**Affiliations:** a Guangxi Forestry Research Institute, Nanning, China; b Guangxi State-Owned Qipo Forest Farm, Nanning, China; c Guangxi State-Owned Huangmian Forest Farm, Liuzhou, China

**Keywords:** Chloroplast genome, Tetrastigma planicaule, Phylogenetic analysis

## Abstract

We announce here the first complete chloroplast genome sequence of *Tetrastigma planicaule*, one important Chinese folk medicinal plant. This complete chloroplast genome is 160,323 bp in length. In total, 131 genes were identified, including 86 protein-coding genes, 37 tRNA genes, and 8 rRNA genes. The results of phylogenetic analysis indicated that *Tetrastigma* is a separate genus and is closely related to *Vitis.*

*Tetrastigma planicaule* (Hook.) Gagnep. belongs to genus *Tetrastigma* (Miq.) Planch. (Vitaceae). It is a wild evergreen woody liana, and is mainly distributed in China, India, Laos and Vietnam (Ren et al. 2007). *Tetrastigma planicaule* is an important Chinese folk medicinal plant which mainly used in the treatment of rheumatoid arthritis, lumbar muscle strain, urticaria and asthma (Chen 2017; Shao et al. 2010). It is also an excellent ornamental plant because of its flat stems and the characteristic of flowering and fruiting on old stems (Zhang et al. 2012). Here, we assembled and characterized the complete chloroplast genome of *T. planicaule*, which would enrich the gene information and contribute to further study of this plant.

The plant material of *T. planicaule* was collected from county Daxin (Chongzuo, Guangxi, China; 22.31°N, 106.80°E). Voucher specimens were deposited at the herbarium of Guangxi Forestry Research Institute (Mr Li, zzcx_gfri@163.com) under the number 2020120401, and DNA samples were stored at Guangxi Key Laboratory of Superior Timber Trees Resource Cultivation, Nanning, China. Raw data were generated using the NexteraXT DNA Library Preparation Kit (Illumina, San Diego, CA) with 150 bp paired-end read lengths, then raw sequence reads were edited using the NGS QC Tool Kit v2.3.3. High-quality reads were assembled into contigs using the denovo assembler SPAdes 3.11.0 software (Bankevich et al. 2012). Finally, the complete chloroplast genome was annotated by PGA software (Qu et al. 2019) and submitted to GenBank under the accession number of MW401672.

The total length of complete chloroplast genome of *T. planicaule* was 160,323 bp, with a total GC content of 37.49%. The complete chloroplast comprised of a large single-copy (LSC) region of 88,181 bp, a small single-copy (SSC) region of 19,096 bp and two inverted repeat (IRS) regions of 26,523 bp. A total of 131 genes were contained in the complete chloroplast genome, including 86 protein-coding genes, 37 tRNA genes and 8 rRNA genes.

Eleven complete chloroplast genomes of other species were selected to confirm the phylogenetic position of *T. planicaule*, with *Cinnamomum camphora* and *Magnolia lilifolra* used as outgroups. All of these complete chloroplast sequences were aligned by the MAFFT version 7.429 software (Katoh and Standley 2013) and trimmed by TrimAl (Capella-Gutierrez et al. 2009). A maximum-likelihood (ML) tree was inferred by ultrafast bootstrapping with 1000 replicates through IQ-TREE 1.6.12 (Nguyen et al. 2015) based on the TVM + F + R2 nucleotide substitution model, which was selected by ModelFinder (Kalyaanamoorthy et al. 2017) . The result of phylogenetic analysis indicated that *Tetrastigma* is a separate genus and it is closely related to *Vitis* (Figure 1)*. Tetrastigma* and *Vitis* were proved to be sister groups through plastid rbcL DNA sequence (Ingrouille et al. 2002). Our study makes this phylogenetic relationship more clearly, and the results are consistent with previous studies of Yu et al (2019).

**Figure 1.**
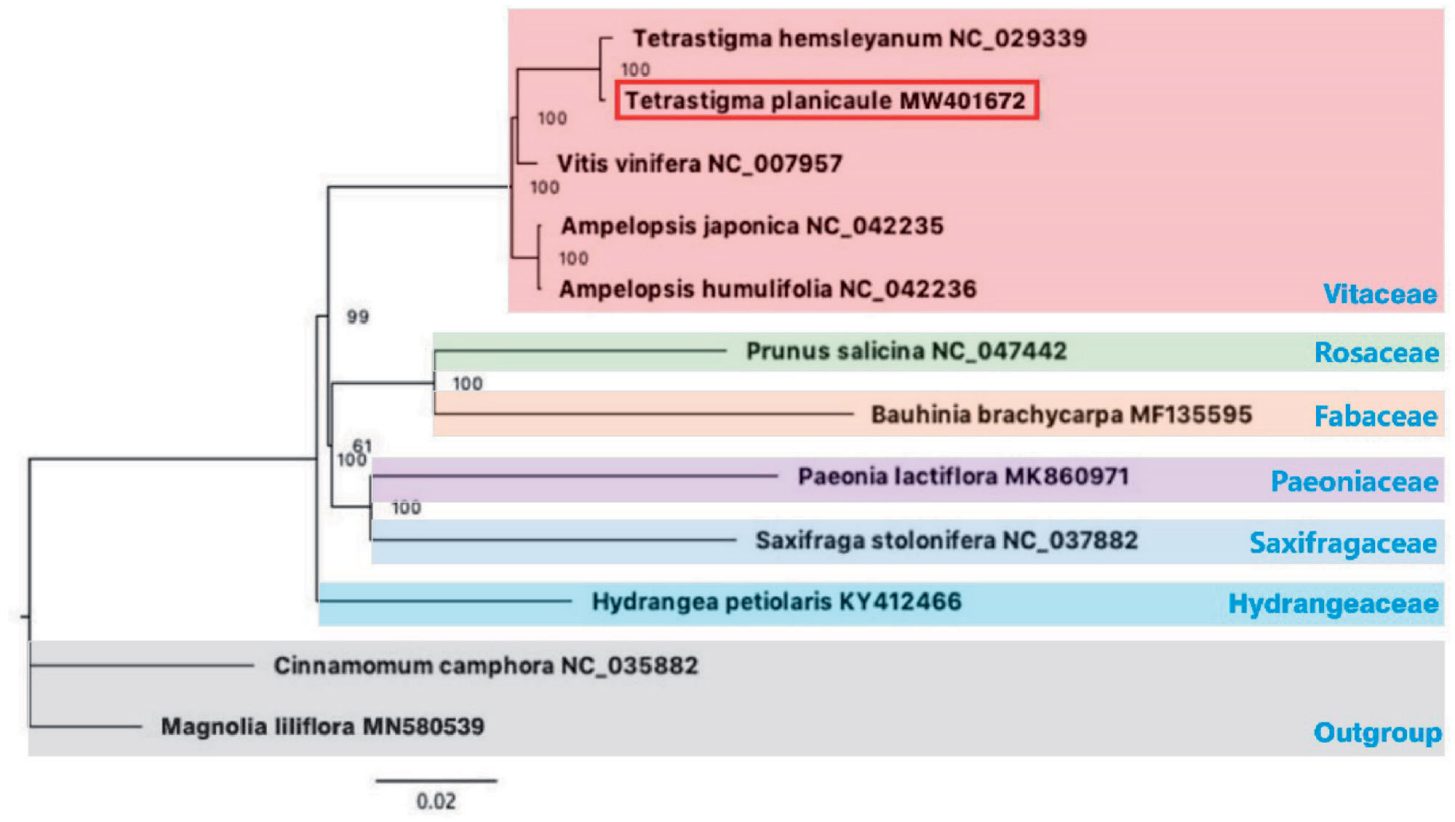
The ML phylogenetic tree based on the complete chloroplast genomes of *Tetrastigma planicaule* and other 11 species. Numbers near the nodes represent ML bootstrap value.

## Data Availability

The data that support the findings of this study are openly available in GenBank number MW401672 (https://www.ncbi.nlm.nih.gov/nuccore/MW401672.1/) and SRA number PRJNA715476 (https://www.ncbi.nlm.nih.gov/sra/PRJNA715476).
